# Contributions of immune cell populations in the maintenance, progression, and therapeutic modalities of glioma

**DOI:** 10.3934/allergy.2018.1.24

**Published:** 2018-03-21

**Authors:** Michael D. Caponegro, Jeremy Tetsuo Miyauchi, Stella E. Tsirka

**Affiliations:** Department of Pharmacological Sciences, BioMedical Sciences, Stony Brook University, Stony Brook, NY, USA

**Keywords:** glioma TME, immunotherapy, T Cells, GAMs, neuropilin 1

## Abstract

Immunotherapies are becoming a promising strategy for malignant disease. Selectively directing host immune responses to target cancerous tissue is a milestone of human health care. The roles of the innate and adaptive immune systems in both cancer progression and elimination are now being realized. Defining the immune cell environment and identifying the contributions of each sub-population of these cells has lead to an understanding of the immunotherapeutic processes, and demonstrated the potential of the immune system to drive cancer shrinkage and sustained immunity against disease. Poorly treated diseases, such as high-grade glioma, suffer from lack of therapeutic efficacy and rapid progression. Immunotherapeutic success in other solid malignancies, such as melanoma, now provides the principals for which this treatment paradigm can be adapted for primary brain cancers. The central nervous system is complex, and relative contributions of immune sub-populations to high grade glioma progression are not fully characterized. Here, we summarize recent research in both animal and humans which add to the knowledge base of how innate and adaptive immune cells contribute to glioma progression, and outline work which has demonstrated their potential to elicit anti-tumorigenic responses. Additionally, we highlight Neuropilin 1, a cell surface receptor protein, describe its signaling functions in the context of immunity, and point to its potential to slow glioma progression.

## 1. Introduction

High grade gliomas (HGG), such as grade IV Glioblastoma Multiform (GBM), are the most common and lethal primary tumors arising in the CNS [1]. GBM are viciously invasive, present chemo- and radio-therapy resistance, are histologically heterogeneous, and more recently have been classified by molecular subtypes [1–4]. Standard therapy minimally increases median survival and involves maximal surgical debulking followed by Temozolomide and radiation treatment regiments [5]. GBM can arise from glial cells throughout the brain, and often results from malignant progression of grade II/III gliomas. The pathological hallmarks of glioma progression parallel those in other malignant solid tumor types, such as the dependency on vascular remodeling and angiogenesis, local tissue invasion, immune evasion, and resistance to standard of care therapies.

Increasing evidence supports the concept that the tumor microenvironment (TME) plays a modulatory role in glioma progression. The TME consists of non-cancerous stromal cell types, all of which ultimately contribute to maintenance and health of the bulk tumor [6]. While the CNS was once believed to be immune privileged, peripheral immune influences on CNS diseases is now a pragmatic subject. Here we discuss how major immune cell populations contribute to the progression and maintenance of HGG, and outline their potential to mitigate the advancement of disease. Lastly, we discuss Neuropillin 1 (NRP1), a prominent cell surface protein receptor with many distinct ligands, as a potential therapeutic target across immune cell populations, and suggest that NRP1 could be exploited in developing new treatments for GBM.

## 2. Discrete roles of immune cell populations in glioma progression, maintenance, and regression

### 2.1. Subpopulations of lymphocytes affect glioma progression

Although lymphoid populations vary across GBM cohorts, increased tumor infiltrating lymphocytes (TILs) correlate with glioma grade, but can also correlate with increased survival [7–9]. This paradox may be elucidated by considering the various lymphocytic populations present in the TME, and by identifying their contributions to tumor development. Naive CD4^+^ lymphocytes arise from hematopoietic thymic progenitors and are activated via MHC II antigen presentation on antigen presenting cells (APCs). Antigen exposure and humoral signaling initiate CD4^+^ T cells to expand into a variety of effector subsets. Polarized Th_1_/Th_2_ helper T cells (Th) are canonically derived from IL-12/INFγ or IL-4 exposure, respectively [10]. T regulatory cells (Treg) are another important subset of effector lymphocytes, which are potently induced following TGFβ exposure [11]. CD4+ Th cells have the ability to mount inflammatory responses, as well as activate processes of adaptive immunity. Alternatively, CD4+CD25+FOXP3+ Tregs are classically associated with immunosuppression, attenuation of autoimmunity, and the inhibition of CD4+ proliferation [11]. CD4^+^ cells are required for adaptive immune system activation, and thus, their presence would be expected to correlate with a stronger anti-tumoral adaptive immune response. However, the influences CD4^+^ Th_2_ and CD4^+^FOXP3^+^ Treg cells have within the TME have not been fully clarified in glioma.

Fecci et al. have previously reported that patients with GBM present with CD4+ lymphopenia, but noticeably also maintain higher proportions of CD4^+^CD25^+^FOXP3^+^ regulatory T cells (Tregs) [12]. Treg depletion studies in culture and in an orthotopic murine model of glioma suggested that the activity of the increased hematogenous populations of Tregs in GBM patients may be linked to Th_2_ responses and suppressed proliferation of CD4^+^ T cells [12]. The same group demonstrated that anti-CTLA-4 treatment in mice harboring malignant astrocytomas increased peripheral CD4^+^ cell numbers and conferred resistance to Treg immunosuppression [13]. Since this work, there has been an increased focus on the contributions of CD4^+^ Th cells and subset CD4^+^FOXP3^+^ Tregs to glioma progression at both the pre-clinical and clinical levels.

Following entry into the CNS/tumor compartment, lymphocytes downregulate CD28, and CD62L co-stimulatory molecule expression [8]. This may hint at a mechanism by which the existing immunosuppressive TME captures and represses TILs. Although CD4^+^ cell numbers alone do not directly correlate with clinical outcome in GBM patients [7,14], new evidence suggests that elevated CD4^+^ and/or CD4^+^FOXP3^+^ population *ratios* may be indicative of glioma disease severity and risk of recurrence [15,16].

The mechanisms underlying how Th and Treg populations aid glioma progression have not been fully characterized. Mu et al. conducted an elegant study which analyzed 44 paired samples from patients with recurrent HGG [17]. Elevated numbers of perivascular CD4^+^ TILs strongly correlated with CD34^+^ tumor vascularity in both primary and recurrent glioma [17]. In a subset of patients refractive to bevacizumab anti-angiogenic therapy, increased and activated CD4^+^ populations were found to be correlated with bevacizumab resistance, as such activation was not apparent in chemotherapy- naïve patient samples [17]. Elevated CD4^+^ and CD4^+^FOXP3^+^ populations were correlated with shorter recurrence-free survival, and the perivascular CD4^+^FOXP3^+^ Treg population in primary tumors was identified as an independent predictor of tumor recurrence in this cohort [17]. The close association to the perivascular region, and conspicuous relationship with tumor progression and recurrence may point to the angiogenic process which may contribute to grade III glioma progression to GBM, a mechanism which has long eluded glioma biology. Nevertheless, these data support negative roles of CD4^+^ population subsets in glioma progression.

Despite these supportive roles, selectively modulating CD4^+^ populations could be used to elicit tumor shrinkage. Anti-tumorigenic effector functions of these cells have been realized in mouse models of melanoma and pancreatic cancer [18–20]. In a syngeneic orthotopic murine model of glioma, CD4^+^ depletion completely nullified tumor lysate vaccine/Fc-OX40L treatment efficacy, and the survival effects were found to be driven in part by antibody-dependent cell mediated cytotoxicity (ADCC) and natural killer T cell (NKT) populations [21]. Similarly, CD4^+^ cell populations were found to be necessary for the complete efficacy of combined oncolytic herpes simplex virus (oHSV ΔG47-mIL12) and immune checkpoint inhibitor therapy in two distinct murine derived glioma models [22].

The anti-tumorigenic potential of alternate lymphocytic populations is also supported by a study, which expanded and differentiated glioma patient T cells (of mixed CD3^+^CD4^−^CD8^−^, CD4^+^, and CD8^+^ subsets) *ex vivo* using IL-2, IL-15, and IL-21. The study demonstrated preferential expansion of existing memory effector T cells populations, which were reactive against autologous tumor cells and shared tumor-associated antigens [23]. The authors also suggest that these expanded cell populations were resistant to TME immunosuppressive factors, and proposed this protocol for adoptive cell transfer therapy application [23]. While other specialized T cell subsets, such as γδ T cells, may lack roles in immune-mediated responses to HGG [24], CD4^+^ T cells and CD4^+^FOXP3^+^ Treg subsets cannot be ignored when considering HGG progression and treatment responses.

### 2.2. Challenges cytotoxic lymphocytes face during glioma rejection

Unlike CD4^+^ cells, there is appreciably more knowledge surrounding the mechanisms by which CD8^+^ cytotoxic lymphocyte (CTL) populations affect glioma progression. Stimulated by MHC I^+^ APCs, effector CD8^+^ T cells selectively target virus-infected, malfunctioning, and/or cancerous cells. CTL infiltrate is typically correlated with survival in GBM patients [25]. Consequently, ineffective tumor clearance arises when tumor cells express ligands, which directly inhibit CTL function. Programmed death ligand 1 (PD-L1) is a primary immunosuppressive molecule whose expression is correlated with glioma grade, and may be a prognostic marker of GBM survival [26]. However, it should be noted that PD-L1 expression among GBM subtypes is inconsistent [27]. Tumor cell expression of PD-L1 is a major mechanism by which the TME exerts immunosuppressive effects via ligation with PD-1 on CD8^+^ CTL effector immune cells. Success in phase I/II clinical trials for GBM patients using PD-1 checkpoint inhibitors Pembrolizumab and Nivolumab has demonstrated that PD-1 blockade may be a promising strategy to control glioma progression [28,29]. Animal models of GBM demonstrate that CTL effector function underlies PD-1/PDL-1 blockade responses [30]. However, efficacy of PD-1 blockade may be dependent on tumor PD-L1 expression levels, and the perquisite existence of PD-L1 subdued CTLs [31]. These caveats present interesting challenges when considering PD-1 blockade as a treatment strategy for GBM. As such, combinatorial treatments aim to more effectively stimulate, recruit and prime CTL populations. Supporting this theory, radiotherapy has shown to dramatically increase the efficacy of checkpoint inhibitor blockade in animal models of glioma, which is characterized by increased CTL infiltrate and diminished Treg populations [32,33]. This regimen can easily be applied to human subjects, as radiotherapy is already a component of standard of care for HGG.

Other methods designed to improve CTL effector function utilize adoptive cell transfer (ACT). ACT elicits potent anti-tumor responses via exogenously priming or genetically altering effector CD8^+^ T cells to recognize tumor specific antigens. This allows CTLs to enter the brain parenchyma, identify target cells, clonally expand, and elicit INFγ-dependent, cytotoxic anti-tumorigenic responses [34]. ACT has been successful in targeting primary melanoma and melanoma brain metastases [35,36], however they are still in initial phases of being developed for malignant gliomas. Intracranial and systemic delivery of autologous T cells expressing genetically engineered chimeric antigen receptor (CAR) against the tumor antigen IL13Rα2 has recently been demonstrated to be a tolerable platform to treat patients with advanced GBM [37]. Following this study, a recent individual case has reported compete regression of recurrent, multifocal tumors following modified IL13Rα2 CAR T-cell therapy [38]. Other modalities, such as the identification of a CD8-independent mechanism of tumor regression in a murine model of glioma via Fc-OX40L may shed light on how discrete lymphoid populations contribute to tumor control [21].

Perhaps the most striking advancement in GBM immunotherapy is the application of dendritic cell (DC) vaccines. Native CTLs are often insufficient to induce GBM disease regression, by virtue of the immunosuppressive TME, and due to the majority of unrecognizable surfaces within the bulk tumor. Exogenously introducing professional antigen presenting cells (APCs) which have been pre-educated to tumor antigen profiles can enhanced activation of the adaptive immune system, allowing for subsequent induction and recruitment of sufficient levels of host derived CTLs. This process may then tip the balance in favor of tumor rejection. Although it has been difficult to predict DC vaccination efficacy, the platform has proven to be a promising as well as tolerable approach to increase CTL infiltrate and GBM patient responses [39,40].

DC vaccines are faced with their own pitfalls including insufficient activation of the adaptive immune system, tumor heterogeneity, and limited migration of activated cells. An approach to circumvent the limited priming of the adaptive immune system is to combine DC vaccines with ACT. Tumor RNA-pulsed-DCs cocultured with autologous lymphocytes effectively expand tumor specific CTL populations, and in conjunction with DC vaccination, ACT significantly improves survival in animals with HGG [41]. Further protocols, which expand tumor specific CTLs, may be extended to humans. Tumor antigen-pulsed DCs from HLA-A*02-positive GBM patients can increase CD8^+^ T cell expansion and specificity *ex vivo* [42]. INFγ production is signature to functional CTL effector populations. CTL responses to tumor antigens can be measured by INFγ production as well as effector/target killing ratios, which could help identify potent CTL effector populations for ACT [42].

Deriving DC vaccination efficacy has also been demonstrated by altering the antigenic profile DCs present to the adaptive immune system. Immunogenic cell death (ICD) of glioma cells induced by photodynamic therapy elicited a significantly stronger DC vaccination response over typical DC priming techniques in a prophylactic animal model of glioma [43]. ICD generates reactive oxygen species and damage associated molecular patterns (DAMPs), which drives DCs to confer robust protection and inhibition of glioma progression [43]. Furthermore, ICD based DC vaccination increased brain Th_1_ and CTL infiltrates, INFγ levels, and reduced Treg population ratios following glioma induction [43]. This approach was also found to be synergistic with traditional DC priming techniques, such as glioma cell freeze/thaw necrosis, as well as standard Temozolomide treatment [43]. Thus, new efforts should consider where tumor antigens are derived and by which methods DCs are primed before vaccination, so that complete activation and specificity of CTLs may be produced.

Lack of DC activation and migration also presents an obstacle for DC vaccination. GBM patients receiving intracranial injection of recall antigen tetanus/diphtheria (Td) toxoid to precondition the vaccination site showed improved clinical outcomes following CMV-pp65 RNA pulsed DC vaccine treatment [44]. Patients displayed a robust increase in number of DCs draining into vaccine site lymph nodes. Further investigation identified a CD4^+^ T cell dependent mechanism, which mediated DC recall and Td precondition efficacy [44]. Prompting DC lymph node draining, cell maturation, and adaptive immune communication are all necessary to induce DC vaccine functionality.

### 2.3. Glioma associated microglia and macrophages

Glioma associated microglia and macrophages (GAMs) traffic to malignant lesions, where they become subverted by tumor cells to adopt a pro-tumorigenic phenotype. Although the paradigm of polarized “M1” or “M2” phenotypes has recently been challenged [45], it is widely accepted that GAMS are predominantly M2-like; and orchestrate tumor progression by secreting factors which promote chemoattraction, immune suppression, neoangiogenesis, tumor cell survival, and by influencing extracellular matrix (ECM) reorganization (reviewed in [6,46,47]). GAMs are considered to be an integral part of glioma pathology, and evidence suggests that modulation of this immune cell population could slow or cause regression of tumor growth.

HGG biopsies consistently show excessive GAM infiltrate [9,48,49], and GAM populations reportedly comprise up to 30% of tumor bulk [47]. GAM infiltrate has been correlated with poor prognosis, particularly those which express M2 markers [50–52]. *In silico* and transcriptional analyses of patient samples link excessive M2 GAM infiltrate to the aggressive mesenchymal GBM subtype, and suggest that alterations in the TME promote GAM recruitment and disease progression [53]. It has also been shown that GAMs from GBM patients express high levels of PDL1, and upregulate this immunosuppressive ligand in response to tumor secreted IL-10 [54]. By deeply infiltrating peritumoral and bulk lesions, pro-tumorigenic M2 GAMs largely exert negative influence over human glioma progression.

Consistent with pro-tumorigenic M2 GAMs found in human patient samples, dynamic characterization of immune cell populations and transcriptomic analysis of GAMs in C6 rat gliomas definitively identify accumulation of immunosuppressive CD4^+^ and Treg populations, and high expression of M2 markers [55]. The mechanisms by which GAMs contribute to glioma progression extend from regulating inflammatory responses to neoangiogenesis. Modulating immunosuppressive TGFβ activity through exogenous miRNA delivery can abrogate M2 GAM populations and prolong animal survival [56]. The anti-tumorigenic response in animal models, elicited by modulating GAM populations, is accompanied by significant downregulation of M2 associated genes, including Arginase-1 (Arg1), Adrenomedullin (Adm) and CD206 [57–61]. Suppressing the M2 phenotype is paramount for controlling GAM pro-tumorgenic functionality, however, shifting this innate immune cell population towards an M1 phenotype may be an additional mechanism to stifle glioma progression. GAM specific SOCS3 KO cells upregulate the JAK/STAT signaling pathway in conjunction with increased pro-inflammatory markers TNFα and CXCL10 [57]. M1 shifted GAMs were shown to induce anti-tumorgenic responses by altering the immune landscape through increasing CD8^+^ populations while simultaneously decreasing Treg populations, although, only mild improvements in animal survival and tumor burden were observed [57]. This presents the notion that GAMs within the TME may be persuaded to shed their subverted pro-tumorigenic phenotype and adopt more anti-tumorigenic behaviors.

Indeed, this idea has demonstrated effectiveness in the context of colony stimulating factor 1 receptor (CSF1R) inhibition, where GAMs can become “re-educated” within the glioma microenvironment to adopt an anti-tumorigenic phenotype [58]. Further, CSF1R inhibition using the experimental compound PLX3397 has been shown to reduce disease progression and M2 gene signatures in preclinical models of glioma, and has enhanced efficacy over the broader spectrum tyrosine kinase inhibitors Vatalanib and Dovitinib [59]. PLX3397 is currently in active clinical trials for malignant solid tumors including recurrent GBM. More recently, CSF1R inhibition has shown to prevent resistance to anti-VEGF therapy in orthotopic model using ovarian cancer cells [62]. These studies highlight CSF1R as a major target by which GAM contributions to glioma progression can be controlled.

**Figure 1.**
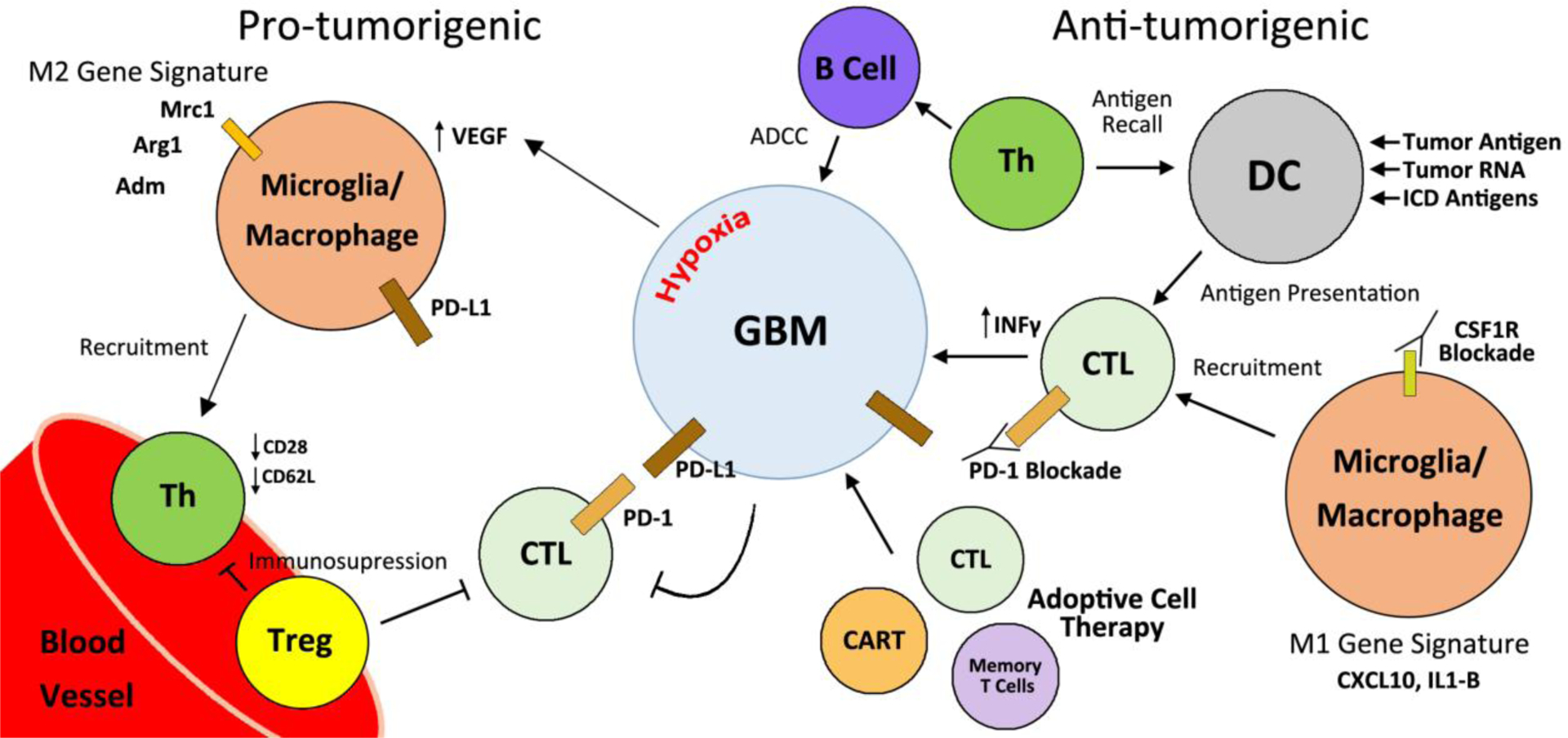
Contributions of immune cell populations in the maintenance, progression, and treatment options of glioma. Perivascular association of CD4^+^ Th and Treg correlates with glioma progression and recurrence. Treg populations are responsible for immunosuppressive effects within the TME and the periphery. CTLs are inhibited within the TME by relatively high Treg populations and PD-L1 ligation. M2 GAMs recruit immunosuppressive T Cells, express PD-L1, and contribute to the VEGF mediated angiogenesis feedback loop. Conversely, DC vaccines, adoptive cell therapy techniques, and GAM manipulation can modulate the immune landscape to exert anti-tumorigenic responses.

Hypoxic tumor regions also induce potent angiogenic signaling in tumor associated macrophages (TAMs). This process regulates the expression of VEGF, contributes to vascular remodeling, and is reportedly dependent on the activity of hypoxia-inducible factor 1-alpha (Hif1α) [60,63]. Additionally, *in vitro* experiments demonstrate that M2 macrophages are responsible for inducing angiogenesis [64]. Thus, regulating GAM populations within the TME could provide a valid method to control immunosuppression and aberrant angiogenesis associated with HGG (Figure 1).

GAMs are potent sources of secreted chemokines, which drive immunosuppressive TIL recruitment. Chemokines, such as CCL2, have been correlated with TIL levels as well as decreased GBM patient survival [65]. To investigate this possible connection, Chang et al. utilized *in vitro* and *in vivo* systems in the GL261 murine glioma model and reported that soluble factors produced by tumor cells induce Arg1^+^ M2-like GAMs within the TME to secrete high levels of CCL2. CCL2 production correlated with distinct CCR4^+^ Treg and CCR2^+^ myeloid derived suppressor cell (MDSC) populations infiltrating the tumor, suggesting these molecular steps may be largely responsible for the recruitment of immunosuppressive cell types in glioma [65]. Other chemokines also play roles in local and peripheral immune recruitment during glioma progression. Activated microglia associated with NF1 low-grade optic gliomas were found to express significantly higher levels of CCL5 and CXCL13 RNA [66]. Antibody mediated CCL5 blockade reduced glioma growth and decreased microglia recruitment to tumor cells, indicating that this chemokine has local CNS effects and enhances the TME growth supporting functions [66].

Contribution to chemotaxis is not limited to GAMS; transplanted hematopoietic stem cell (HSC) prior to ACT in an animal model of glioma were shown to be necessary for lymphocyte recruitment and effective tumor rejection [41]. The chemoattracting properties of the HSCs, specifically the secretion of CLL3, was determined to be the governing factor for ACT efficacy [41], supporting the roles that immune signaling proteins have in glioma maintenance and progression. Thus, factors produced both by the tumor as well as immune cells of the TME contribute to the remodeling of the immune landscape. Cell signaling profiles may have important implications when considering how patients will respond to therapies.

Neuropilin 1 has recently been identified as a receptor involved in the activation of GAMs [67,68]. Our group has demonstrated that binding of the immunomodulatory tetrapeptide, tuftsin (TKPR), to Nrp1 in the setting of experimental autoimmune encephalomyelitis (EAE), a rodent model of Multiple Sclerosis, has the potential to polarize microglia to a more immunosuppressive phenotype via TGFβR1 and SMAD2/3 activation, thereby reducing the severity of the disease course [67]. This initial observation led to the examination of Neuropilin’s potential role in the immune regulation of glioma microenvironment.

## 3. Neuropilin 1: an immunotherapeutic, anti-proliferative, and anti-angiogenic target for glioma

Neuropilin 1 (Nrp1) is a cell surface receptor which was originally identified to contribute to signaling associated with axonal pathfinding and chemorepulsion via co-reception with one of its associated co-receptors, Plexin A1, in neurons [69]. Nrp1 has since been found by various groups to also have the potential to complex with other co-receptors including transforming growth factor β receptor I/II (TGFβRI/II), vascular endothelial growth factor receptor 2 (VEGFR2), hepatocyte growth factor receptor (cMET) and to amplify signaling pathways associated with these receptors [70–72].

**Figure 2.**
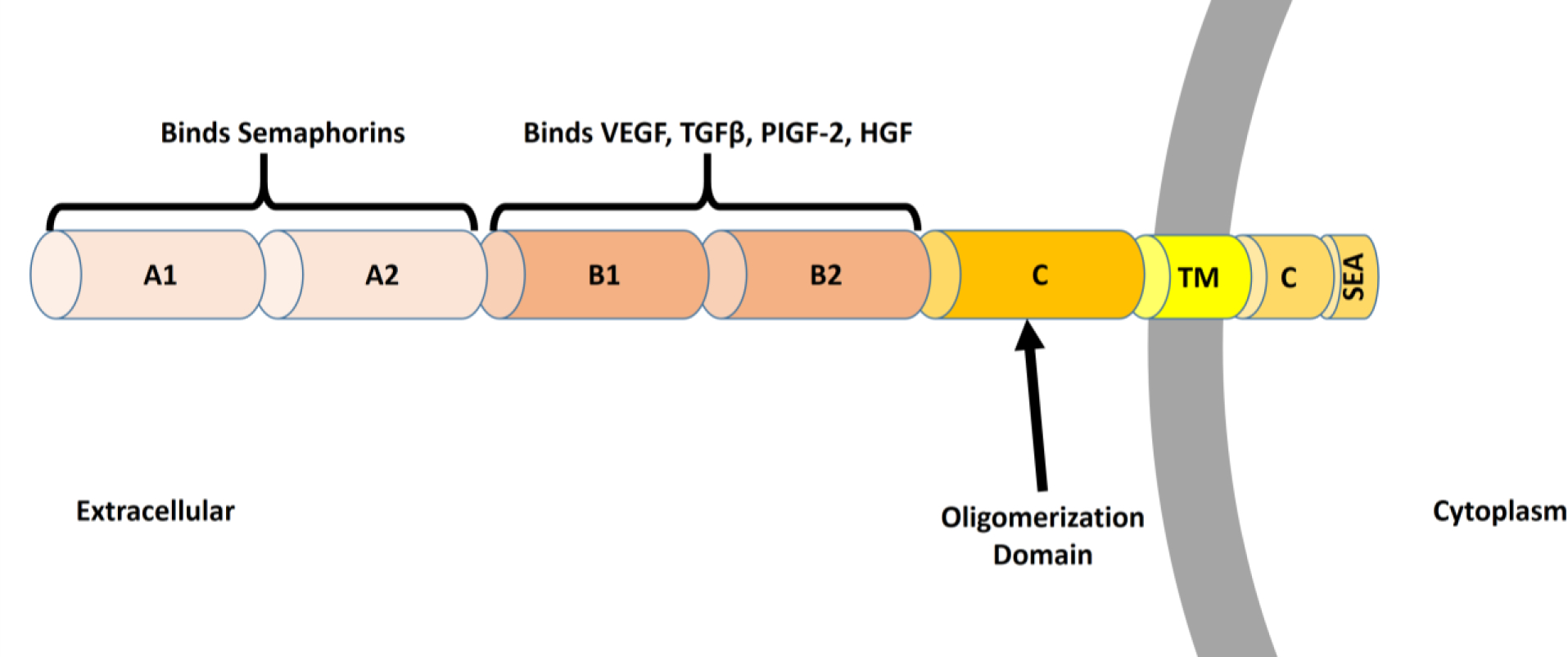
Structure and Function of Neuropilin 1. Neuropilin 1 (Nrp1) is a cell surface receptor composed of an A1, A2, B1, B2, C, transmembrane (TM), and a C-terminal (C) domain. The C-terminal domain contains a SEA motif which binds PDZ adaptor proteins. The A1 and A2 domains are responsible for binding semaphorins while the B1 and B2 domains have been characterized to bind VEGF, TGFβ, PlGF, and HGF. The C domain is an oligomerization domain, which allows Nrp1 to interact with its various co-receptors and amplify signaling via their associated ligands.

Nrp1 is composed of an A1, A2, B1, B2, oligomerization, transmembrane, and cytoplasmic tail domain. The cytoplasmic tail domain is quite short and is considered to have no potential to elicit downstream signaling on its own (Figure 2). However, in complex with an associated receptor via interactions with its oligomerization domain, Nrp1 has the potential to amplify the associated receptor’s signaling pathway [73]. The A1 and A2 domain are semaphorin binding domains while the B1 and B2 domains are responsible for binding VEGF, TGFβ, PIGF, and HGF [74]. Additionally, it has been shown that Nrp1 can complex with ABL1 in endothelial cells and carry out angiogenic signaling independently of its association with VEGFR2 or VEGF [75].

Nrp1’s expression is rather ubiquitous in terms of its tissue distribution in that it is expressed by endothelial cells, subsets of DCs, subsets of T cells, subsets of myeloid-derived cells, and microglia [74–76]. In mice and rats, the complete elimination of Nrp1 is lethal due to its crucial role in embryonic angiogenesis [77,78]. In a similar manner, mice which express Nrp1 with a point mutation in the B1 domain responsible for signaling via TGFβ, PlGF, HGF, and VEGF-A, survive to adulthood but exhibit abnormal vasculature [78]. These mice have been shown to have a resistance phenotype to the growth of xenograft tumors, attributed to poor neovascularization of the tumors [78].

### 3.1. Functional roles of NRP1 in T cells

Nrp1 is expressed by subsets of Tregs and plays a role in the suppression of adaptive immunity [76]. In skin allograft experiments in mice, it was seen that the survival of the tissue was partially dependent on the expression of Nrp1 by Treg populations. When this expression was lost, the allografts were rejected, hinting at an immunosuppressive role for Nrp1 in these cells [79]. NRP1 may also function to suppress autoimmunity, as CD4^+^ loss of NRP1 in a mouse model of multiple sclerosis skews inflammatory populations to a TH-17 phenotype and reduces Treg populations, worsening autoimmune disease progression [80]. It has also been shown in a murine melanoma model that Nrp1 expressing Tregs are attracted to tumors via the tumor’s secretion of VEGF, which is abrogated by the inhibition of Nrp1 signaling. Delgoffe et al. have shown that the stability of Nrp1^+^ Tregs in the tumor microenvironment is dependent on their activation by the Nrp1 ligand, semaphorin 4A (Sema4A). Antibody-mediated blockade of sema4A or genetic deletion of Nrp1 from these Treg populations using FOXP3-Cre resulted in enhanced anti-tumoral immunity in melanoma mouse models [81]. The accumulation of Nrp1 expressing Tregs in tumors is correlated with increased immunosuppression and the suppression of effector T cell functions [82].

Nrp1 expression in the majority of peripheral T cells is rather negligible in healthy people. However, in patients with certain advanced stage cancers, such as pancreatic adenocarcinoma and colorectal cancer, Nrp1 expressing T cells are significantly elevated in their blood and have been considered as potential biomarkers for their degree of immunosuppression in these patients [74]. Additionally, the expression of Nrp1 has also been consistently documented in naïve populations NKT cells in humans, but its expression is lost in mature populations of the cells [83].

### 3.2. Dendritic cells and innate immune cells

Immature plasmacytoid dendritic cells (iDCs) are another PBMC that has consistently been identified to express high levels of Nrp1 in humans. These cells have the potential to preferentially interact with Nrp1-expressing Tregs via the homotypic interaction of Nrp1 on both cell types, leading to the activation and expansion of these Treg populations, providing an explanation for how they can lead to increased immunosuppression. This “glue” between both cell types has also been postulated to contribute to greater sensitivity of these cells to antigen presentation [84]. Additionally, it has been shown that human Nrp1-expressing DCs have the potential to transfer Nrp1 to Tregs via trogocytosis along with VEGF and potentially other intracellular contents [85].

### 3.3. NRP1 functionality in monocytic populations

Macrophage-specific depletion of Nrp1 in mice via the use of LysM-Cre does not result in any apparent abnormalities in development or in adulthood [86]. However, it has been shown that mice with Nrp1 depletion from LysM-Cre expressing microglia and macrophages are resistant to pathological angiogenesis in a model of retinal sclerosis [87]. These same mice with Nrp1-deficient LysM expressing cells were shown to have slower disease courses in orthotopic breast and pancreatic cancer models, attributed to poorer vascularization of the tumors and increased infiltration of tumors by anti-tumorigenic macrophages and T cells [86]. Our group has reported the expression of Nrp1 by glioma associated microglia and macrophages (GAMs) associated with glioma biopsies of various grades [68]. Additionally, Zhang et al. reported that subsets of highly aggressive gliomas are populated by GAMs with significantly elevated Nrp1 expression [88].

As mentioned earlier, activation of Nrp1 by tuftsin during EAE resulted in polarization of microglia to a more M2-like phenotype via SMAD2/3 activation [67]. We have also observed that Nrp1 depletion from GAMs slows tumor progression and increases anti-tumoral immunity in a murine model of GBM [68]. The outcomes in these diseases models were partially attributable to the fact that Nrp1 complexes with TGFβRI/II to potentiate signaling via SMAD2/3, highlighting its potential as a therapeutic target in a similar fashion to the use of TGFβ inhibitors [89]. Additionally, using chimeric mouse models, we have shown that NRP1 ablation from either populations of peripheral monocytes or resident microglia can repress glioma progression, suggesting discriminant functionality of these cells [90].

### 3.4. Glioma-derived cancer cells

Overexpression of Nrp1 by cancerous cells in glioma biopsies has been directly correlated with poorer clinical outcome and worse progression free survival (PFS) [91]. Various studies have implicated most of the soluble factors known to signal via Nrp1 to be associated with poorer clinical outcomes and direct promotion of glioma growth in animal studies. Chen et al. demonstrated that blocking Nrp1 using a monoclonal antibody inhibited the proliferation and migration of the human-derived glioma cell line U87MG and slowed tumor progression *in vivo* when the cell line was xenografted in mice [92]. Additionally, the U87MG cell line has a highly invasive phenotype relative to glioma cell lines such as the LN18, T98, and U118 human glioma cell lines. *In vitro* analysis of the secretome of the U87MG cell line showed significant elevations in the amount of Nrp1 secreted by the cells relative to the less invasive lines [93] In the U373MG human glioma cell line, it was shown that siRNA-mediated knockdown of Nrp1 reduced proliferation and increased apoptosis of the cells, associated with reductions in Bcl-2 expression and ERK, JNK, and MAPK activation [94]. Additionally, Nasarre et al. showed that targeting the transmembrane domain of Nrp1 via a small peptide inhibitor has the potential to slow glioma progression due to reductions in angiogenesis and proliferation in pre-clinical human xenograft and rat models [95].

### 3.5. NRP1 signaling in glioma maintenance and progression

Semaphorin 3A (sema3A) has been implicated to promote the infiltration and spread of glioma-derived cells in an autocrine fashion and is overexpressed in a subset of gliomas in patients [96]. Sema3A is secreted by glioma-derived cells in vesicles, which have been shown to directly increase vascular permeability by interacting with Nrp1 on endothelial cells in xenograft mouse models. Blocking signaling via either sema3A or Nrp1 was shown to abrogate this. Additionally, these vesicles can be detected in the blood of patients, which may also hold some prognostic value [97]. The expression of the receptor for sema3A, PlexinA1, has been correlated with worse survival outcomes in patients with GBM. Additionally, in a murine xenograft model, it has been shown that a small peptide inhibitor that disrupts the oligomerization of Nrp1 and PlexinA1 reduces GBM proliferative potential and tumor angiogenesis *in vivo* [98]. This peptide inhibitor, interestingly, blocked VEGF-dependent angiogenesis *in vitro* as well, possibly by also blocking the oligomerization of Nrp1 with VEGFR2. Additionally, Casazza et al. have shown that Sema3A acts a chemoattractant for Nrp1-PlexinA1 expressing TAMs to infiltrate tumors where they downregulate Nrp1 expression once becoming entrapped in more hypoxic environments. Deletion of Nrp1 or mutating the sema3A-binding A1 domain of Nrp1 from TAMs was shown to prevent this [86]. As mentioned above, Nrp1 also binds and signal via Sema4A, playing an important role in the maintenance of immunosuppressive T_reg_ populations.

VEGF-A and VEGF-B bind Nrp1 in complex to VEGFR2 and amplify pro-angiogenic signaling via the activation of AKT and p38 MAPK [99]. Glioma stem cells have also been shown to secrete VEGF-A, which not only serves to promote angiogenesis, but also enhances the proliferative index of glioma cells in an autocrine fashion via VEGFR2 in complex to Nrp1 [100]. VEGF-A overexpression is well documented in almost all cases of HGG and has received a great deal of attention as a therapeutic target in recent years. Phase III clinical trials were performed in 2014, evaluating concomitant Avastin (bevacizumab, an anti-VEGF antibody) with TMZ and RT as first line defense for newly diagnosed glioma. While increasing PFS significantly, the trials failed to meet pre-defined criteria for success and failed to show any increase in overall survival time (OST) for patients [101]. However, individual patients responded quite well, showing that some may serve to benefit from the adjuvant therapy. Bevacizumab is still under evaluation as a concomitant therapy in various clinical trials for HGG.

Nrp1 has been shown to bind TGFβ and LAP-TGFβ and amplify signaling associated with these ligands in cancer cells via co-reception with TGFβRI/II [89]. TGFβI and II overexpression, especially that of isoform II, has been correlated with poorer clinical outcomes in subsets of glioma [102,103]. Autocrine signaling within cancer cells serves to enhance epithelial to mesenchymal transition (EMT) and increases the invasive phenotype of tumor cells. TGFβ potentiates angiogenesis and is an immunosuppressive cytokine, which polarizes T_regs_ and attracts and polarizes immunosuppressive GAMs [104,105]. It can downregulate perforin, granzyme A/B, IFNγ, and FasL expression by CTLs, which are all mediators of CTL-mediated cytotoxicity [106]. Downregulation of the expression of TGFβRII in human xenograft-derived gliomas has been shown to reduce their tumorigenicity [107]. Inhibition of TGFβ-dependent pathways using TGFβRII inhibitors in GAMs has been shown to prevent their immunosuppressive polarization [108]. Blocking TGFβ-mediated signaling using systemically administered neutralizing antibodies was efficacious in slowing glioma progression in immunocompetent mice, partially by preventing the immunosuppressive polarization of GAMs [109]. For the treatment of glioma, clinical trials are ongoing, evaluating the TGFβRI small molecule inhibitor, LY2157299, for efficacy in combination with the standard of care. The drug is generally well tolerated and has shown efficacy in about 20% of patients [110].

Placental growth factor (PlGF) is an important angiogenic factor, which has been shown to bind the B1 domain of Nrp1 and act as a chemo-attractant for GAMs [111,112]. Clinical trials have been conducted using a monoclonal antibody against PIGF for recurrent glioma. The drug has shown acceptable safety profiles, but, unfortunately, the antibody was not shown to have any additive benefit for patients over bevacizumab alone [113]. In medulloblastoma, however, PlGF and Nrp1 are both highly expressed. Inhibition of signaling via either has been shown to slow tumor progression in murine xenograft models [114].

Hepatocyte Growth Factor/Scatter Factor (HGF/SF) is a ligand which binds Nrp1 in its complex to cMET and activates downstream signaling which promotes cell proliferation, angiogenesis, and survival [99]. The overexpression of HGF by glioma cells has been correlated with increased tumor microvascularity, increased tumor grade, and worse prognosis for patients. Downregulation of HGF in human-derived glioma cells was also shown to reduce their proliferative and migratory capacity [115]. Furthermore, Hu et al. demonstrated that Nrp1 expression by a subset of human glioma-derived xenografts potentiated their growth in an autocrine fashion via the amplification of pathways downstream of cMET and HGF [71]. A phase II clinical trial was conducted with rilotumumab, a HGF-blocking antibody, in patients with recurrent GBM, but the antibody showed little efficacy as a monotherapy [116]. However, cabozantinib, a small molecule inhibitor of both cMET and VEGFR2, underwent phase II clinical evaluation in patients with progressive and recurrent GBM, and was reported to result in modest improvements in PFS [117]. The drug is currently under evaluation in combination with standard of care RT and TMZ for newly diagnosed GBM [118]. The HGF-cMET signaling axis is a promising therapeutic target, but it would appear that targeting it in combination to other pathways is preferable. As Nrp1, serves to amplify cMET signaling, a similar rationale for targeting it in HGG is well substantiated.

### 3.6. NrpI as a PET tracer and for drug delivery

As Nrp1 is widely expressed in gliomas, the use of PET tracers that bind Nrp1 has been investigated in murine models. Using F-18 labelled peptides, Wu et al. were able to show that peptides targeting Nrp1 and integrin αvβ3 preferentially bound to glioma tissue [119]. This method may hold promise in monitoring glioma progression in patients. Additionally, more effective drug trafficking to gliomas has been proposed by packaging chemotherapeutics in liposomes coated by Nrp1-binding peptides [120–123]. In a similar fashion, targeting Nrp1 in order to deliver gadolinium oxide for MRI and chlorin for interstitial photodynamic therapy has been explored in rat xenograft studies. The nanoparticles were able to preferentially localize to peripheral tumoral vasculature and may hold some promise for translation to the clinic [124].

## 4. Conclusions

Identifying the contributions of immune cell populations within the TME will further the knowledge base by which we treat and develop therapies for GBM. The innate and adaptive immune systems are complex, multifaceted schemes. Providing protection from foreign pathogens, materializing sustained immunity, and regulating self/non-self-responses are immense tasks. Unfortunately, in scenarios of malignancy, the immune system often fails to protect the host. Modulating lymphocyte inflammatory responses may prove to be a method by which overall adaptive immunity can be coerced into rejection of bulk tumors. Additionally, with the advancements of ACT and DC vaccines, tools now exist to selectively activate effector cells of the adaptive immune system. Innate immune cells such as microglia and macrophages are now also recognized as pertinent players to glioma progression, and perhaps by invoking their phagocytic and pro-inflammatory functions, a greater foothold can be gained in controlling HGG disease (Figure 1). New targets which can modulate subsets, or entire arms, of the immune system need to be identified so that clinicians can combat GBM.

Directly targeting Nrp1 in the clinic has only been approached, thus far, for the treatment of advanced solid tumors via the use of a humanized monoclonal antibody, MNRP1685A, which blocks the binding of VEGF-A, VEGF-B, and PlGF-2 to the B1 domain of Nrp1. The antibody was hypothesized to benefit patients through a mechanism similar to bevacizumab, but was unfortunately poorly tolerated and associated with clinically significant levels of proteinuria in patients during phase I evaluation [125].

**Figure 3.**
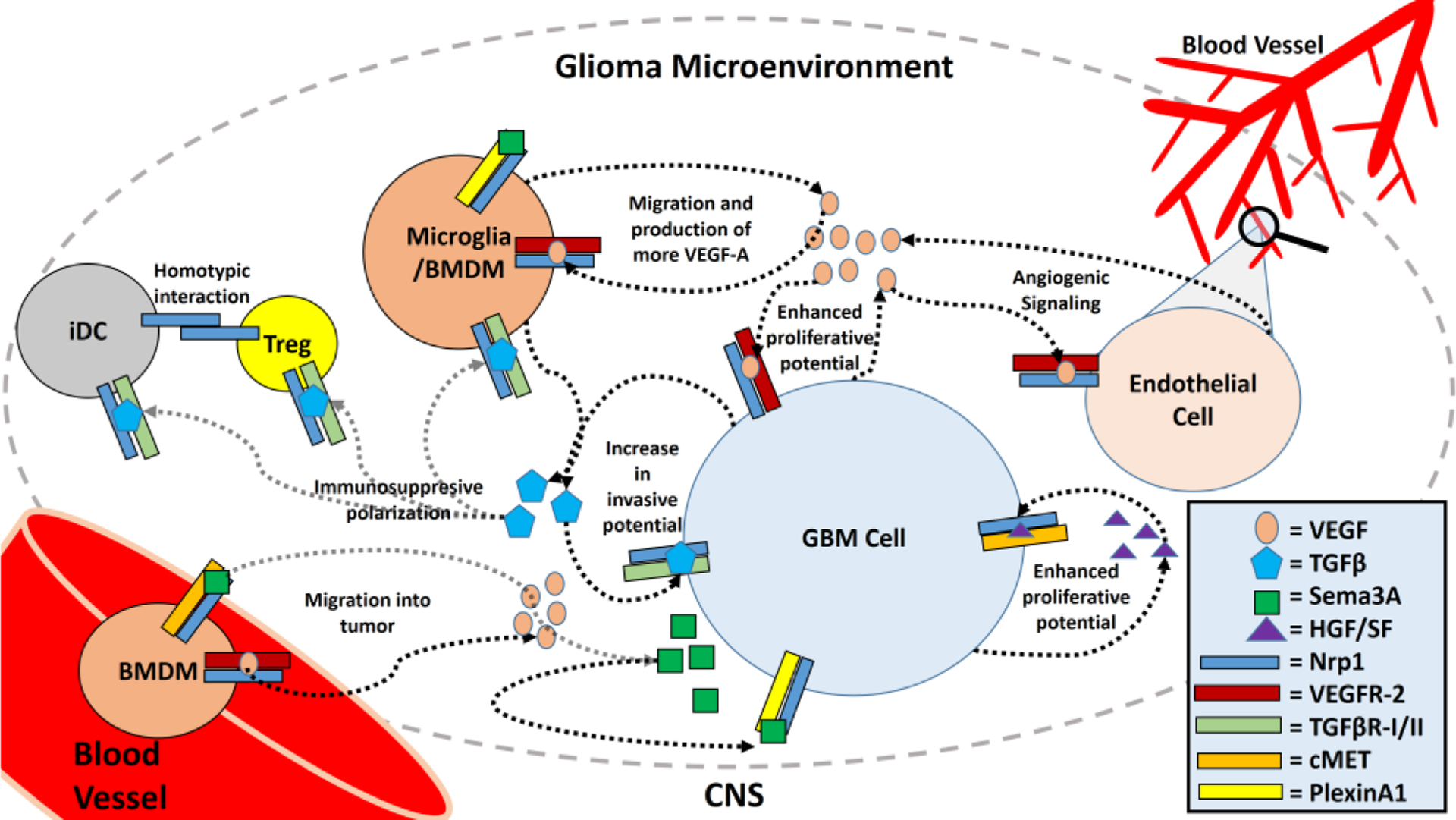
Roles of Neuropilin 1 in the Glioma Microenvironment. Neuropilin 1 (Nrp1) is expressed by various cell types which infiltrate the glioma microenvironment, including most glioma-derived cells (GBM cells), microglia, infiltrating BMDMs, endothelial cells, certain T_reg_ subtypes, and certain dendritic cell subtypes (iDCs). GBM cells produce VEGF, TGFβ, and HGF/SF which increase the malignancy of the tumors by enhancing the proliferative and invasive potential of the GBM cells, mediated via Nrp1 and its associated co-receptors. Sema3A in the glioma microenvironment also causes microglial and BMDM migration into the tumor and enhances invasion by the GBM cells. VEGF produced by the tumors also serves to enhance angiogenesis and increase microglial and BMDM migration into the tumor. Microglia and BMDMs are also responsible for the production of VEGF and TGFβ. TGFβ can polarize microglia, BMDMs, T_regs_, and iDCs to more immunosuppressive, tumor supporting phenotypes. Homotypic interactions between Nrp1 on iDCs and T_regs_ also enhances their contact times and allows for stronger stimulation of these immunosuppressive T_reg_ subsets.

The efficacy and tolerability of other Nrp1-targeting drugs should be considered, and since Nrp1 plays so many roles in the glioma microenvironment (Figure 3), pursuing research in the development and implementation of Nrp1 antagonists in glioma therapy seems fruitful.
